# Effectiveness of lifestyle interventions for treatment of overweight/obesity among children in China: A systematic review and meta-analysis

**DOI:** 10.3389/fendo.2022.972954

**Published:** 2022-10-31

**Authors:** Bo Li, Shan Gao, Wei Bao, Ming Li

**Affiliations:** ^1^ Department of Endocrinology, National Health Commission (NHC) Key Laboratory of Endocrinology, Peking Union Medical College Hospital, Peking Union Medical College and Chinese Academy of Medical Sciences, Beijing, China; ^2^ Department of Endocrinology, Xuanwu Hospital, Capital Medical University, Beijing, China; ^3^ Institute of Public Health, Division of Life Sciences and Medicine, University of Science and Technology of China, Hefei, Anhui, China

**Keywords:** lifestyle interventions, pediatric obesity, childhood overweight, treatment, randomized controlled trials

## Abstract

**Background:**

Pediatric obesity has become a global public health problem. China has the largest population of obese children in the world. It is important to develop effective interventions to control child obesity. This systematic review summarizes proof from randomized controlled trials to assess the efficacy of lifestyle intervention to reduce BMI and metabolic risk factors for overweight/obese Chinese children.

**Methods:**

We searched studies from five databases (PubMed, MEDLINE, EMBASE, Cochrane Database of Systematic Reviews, and CNKI). Research that meets the following requirements was included: randomized controlled trials, participants are overweight/obese Chinese children aged <18 years old, and the effectiveness of lifestyle interventions is evaluated.

**Results:**

Eight randomized controlled trials were included. A significant difference was found between the intervention group and the control group for the BMI changes (mean difference = −1.49; 95% CI: −2.20 to -0.77; p < 0.001). Subgroup analyses consistently demonstrated that effects of lifestyle interventions on BMI change including multiple-component interventions (mean difference = −2.03 kg/m^2^; 95% CI: −3.62 to -0.43; p < 0.001) were stronger than those only with physical activities or health education; effects of lifestyle interventions on BMI change were also strengthened if duration of intervention last for more than 1 year (mean difference = −3.03 kg/m^2^; 95% CI: −4.00 to -2.06; p = 0.01) or with age during 12-18 years old (mean difference = −1.90 kg/m^2^; 95% CI: −3.37 to -0.43; p < 0.001).

**Conclusions:**

Lifestyle interventions are effective in reducing BMI in Chinese children with overweight/obesity, and the effectiveness is more profound when the lifestyle intervention includes multiple components, lasts longer than one year, and/or is conducted among teens. These findings provide an important evidence base for developing and implementing potentially effective lifestyle interventions for the treatment of overweight/obesity among Chinese children.

## Introduction

Pediatric obesity has become a global public health problem (1, 2). China’s latest census results show that the national prevalence in 2015-2019 is estimated to be 6.8% for overweight and 3.6% for obesity in children younger than 6 years, 11.1% for overweight and 7.9% for obesity in 6–17 years’ children and adolescents (3). Childhood obesity is correlated with metabolic disorders like elevated blood pressure, type 2 diabetes, and metabolic syndrome (3–5), which often persist into adulthood (5). Therefore, it is critical to manage and treat overweight and obesity in children.

Lifestyle interventions are the first-line treatment for childhood obesity (6). Several types of interventions, including physical activity (PA), dietary improvement (DI), and health education (HE) have been applied to address overweight and obesity in children (7–9). However, evidence of potential efficacy applies mostly to children in western countries (6, 10, 11), the efficacy and feasibility of lifestyle interventions in children and adolescents in China lack evidence, particularly from systematic reviews, which hinders the development of childhood obesity management in China. It is vital to assess the changes in cardiovascular metabolic outcomes which are closely related to changes in body weight or BMI (12, 13).

Systematic review and meta-analysis of randomized controlled trials were carried out to evaluate the effectiveness of lifestyle intervention for the treatment of overweight/obesity in children in China and to examine the characteristics of intervention components associated with the improvement of metabolic outcomes. Expected results may help children maintain a healthy weight. We speculated that lifestyle intervention improves metabolic outcomes for Chinese overweight/obese children.

## Materials and methods

### Literature search

We searched the five databases (PubMed, MEDLINE, EMBASE, Cochrane Database of Systematic Reviews, and CNKI) to identify RCTs of lifestyle interventions for overweight/obesity among children in China. Both English and non-English language publications between January 1980 and April 2022 were included. We conducted the literature search process using the following keywords (Pediatric Obesity OR Obesity in Childhood OR Childhood Onset Obesity OR Child Obesity OR Childhood Obesity OR Adolescent Obesity OR Childhood Overweight OR Adolescent Overweight) AND (Behavior OR Lifestyle OR Suppress Appetite OR Diet OR Exercise OR Running OR Jogging OR Swimming OR Walk OR Education OR Courses) AND (Therapy OR Management OR Treatment OR Intervention OR Adjustment).

Included researches meet the following criteria (1): individual RCT (2), investigating lifestyle interventions in Chinese children with overweight/obesity (3), collecting anthropometric data through physical examination and calculating body mass index (BMI) (4), providing a brief description of the intervention group and comparison group (5), the full-text publications available. We excluded studies that did not report BMI changes or any metabolic outcome changes.

### Outcomes

The main results were changes in BMI. Secondary outcomes were metabolic outcomes including changes in fasting blood glucose (FBG), blood pressure, total cholesterol, low-density lipoprotein [LDL], high-density lipoprotein [HDL], and triglyceride [TG].

### Data extraction and the risk of bias evaluation

We carefully read the reports of each study, including the intervention and the control group measures, the participants’ numbers, the participant age, the trial period, and the metabolic outcomes. Cochrane risk of bias tools was applied to evaluate the methodological quality of RCT (14, 15). Review manager 5.4 was used to create a visual representation of the results of the risk of bias evaluation.

### Data synthesis

We calculated pooled mean difference (MD) with a 95% confidence interval (CI) for outcomes. We assessed heterogeneity with the I2 test and Chi-square statistics. When heterogeneity across studies was considerable (I2 ≥ 50%), the weighted results are summarized using a random effects model (16). We conducted subgroup analyses to explore the source of heterogeneity. Then, we classified interventions according to specified intervention components (HE, PA, and DI), duration of intervention, and age. Results with P < 0.05 are reported as significant. We performed statistical analysis with the RevMan Version 5.4.

## Results

### Identification and description of inclusion studies

**Figure 1:**
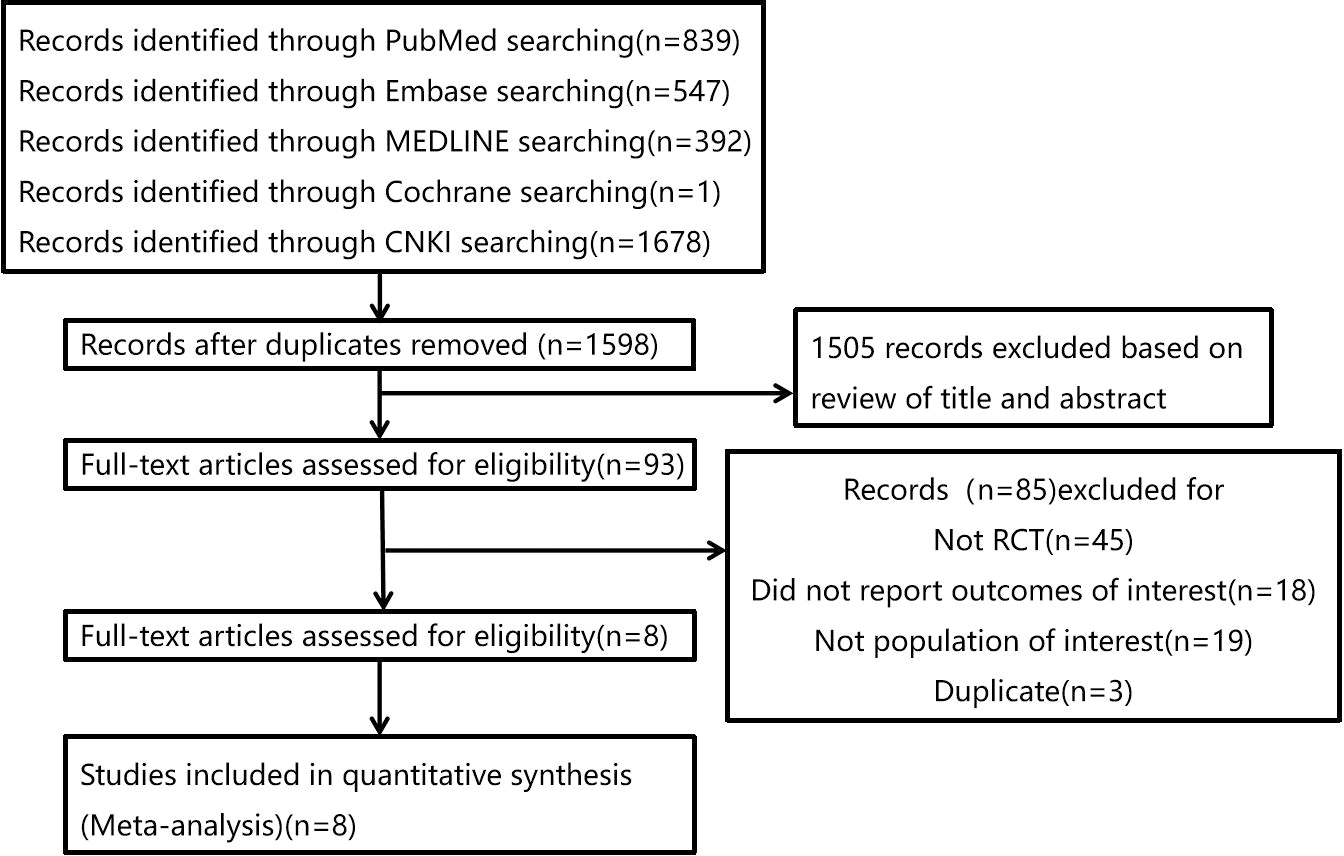
The process of study selection.

**Figure 1** shows our screening process and rational for excluding studies. Eight RCTs were included in the meta-analysis (17–24). Among them, two studies (17, 20) grouped the intervention participants by 3 intervention groups and reported the results separately. Each intervention group has been seen as a single study and analyzed independently. The 12 studies included boys and girls from 6 to 18 years old. All data were extracted from the published papers. **Table 1** reported the characteristics of the included studies. Four studies describing favorable consequences evaluating combined diet and PA interventions (20–22, 24). The other studies showed meaningful intervention effects on BMI, and they have the following characteristics: at least 30-minute moderate exercise each day (n=3), only education sessions (n=2), combined HE and PA interventions (n=2), and record diary behavior (n=1).

**Table 1:** Characteristics of studies on the effects of lifestyle interventions for children with overweight/obesity.

Author, Year	Intervention	Comparison	Patients (intervention)	Patients (comparison)	Age (year)(Mean ± SD)	Intervention duration	Outcomes reported
Hao et al., 2019I (17)	30-minute rope skipping every morning	No intervention	57	56	11.20 ± 0.67	2 months	Change in mean BMI
Hao et al., 2019II (17)	45-minute lecture about nutrition education once a week for 2 months	No intervention	60	56	11.20 ± 0.67	2 months	Change in mean BMI
Hao et al., 2019III (17)	30-minute rope skipping every morning and two 45-minute physical; education classes each week for 2 months	No intervention	56	56	11.20 ± 0.67	2 months	Change in mean BMI
Chen et al., 2018 (18)	The intervention included three major components (1) used a wearable sensor for six months; (2) reviewed eight online health education courses for three months; (3) received text messages for three months.	Record physical activity, sedentary activity, and food intake for three months	23	17	14.90 ± 1.67	6 months	BMI
Chen et al., 2009 (19)	Children participated in a 45- minute session once each week for 8 weeks: 15 minutes for physical activity, 30 minutes for health education	No intervention	35	32	8.97 ± 0.89	2 months	BMI, SBP, DBP
Sun et al., 2011I (20)	15%-20% protein, 25%-30% unsaturated fatty acid, and 50%-60% carbohydrate	No intervention	22	17	13.60 ± 0.70	10 weeks	BMI, TG, TC
Sun et al., 2011I I (20)	60-minute aerobic training, 4 times per week	No intervention	25	17	13.60 ± 0.70	10 weeks	BMI, TG, TC
Sun et al., 2011III (20)	60-minute aerobic training, once a day, 4 days per week; 15%-20% protein, 25%-30% unsaturated fatty acid, and 50%-60% carbohydrate	No intervention	29	17	13.60 ± 0.70	10 weeks	BMI, TG, TC
Jiang et al., 2005 (21)	Exercise for 20-30 minutes per day for four days per week; daily calorie restriction	No intervention	33	35	I:13.30 ± 0.60 C:13.20 ± 0.70	2 years	BP, BMI TG,TC
Luo et al., 2012 (22)	Protein 30%, carbohydrate 50%, and fat 20%; engaged in high-volume aerobic exercise (6 days/week, twice daily, for 3 h per session)	No intervention	95	72	12.10 ± 0.56	6 weeks	BMI, blood pressure, TC, TG, insulin, and glucose
Zhao et al., 2013 (23)	a 40-minute physical activity, 5 times each week for 12 weeks	No intervention	30	15	9.10 ± 0.76	12 weeks	BMI
Zhen et al., 2010 (24)	a 60-minute physical activity, 3 times every week for 12 months + hold good dietary habit	No intervention	30	30	12.40 ± 0.69	12 months	BMI

WC, waist circumference; TG, triglyceride; TC, Total cholesterol.

### Quality evaluation

**Figure 2:**
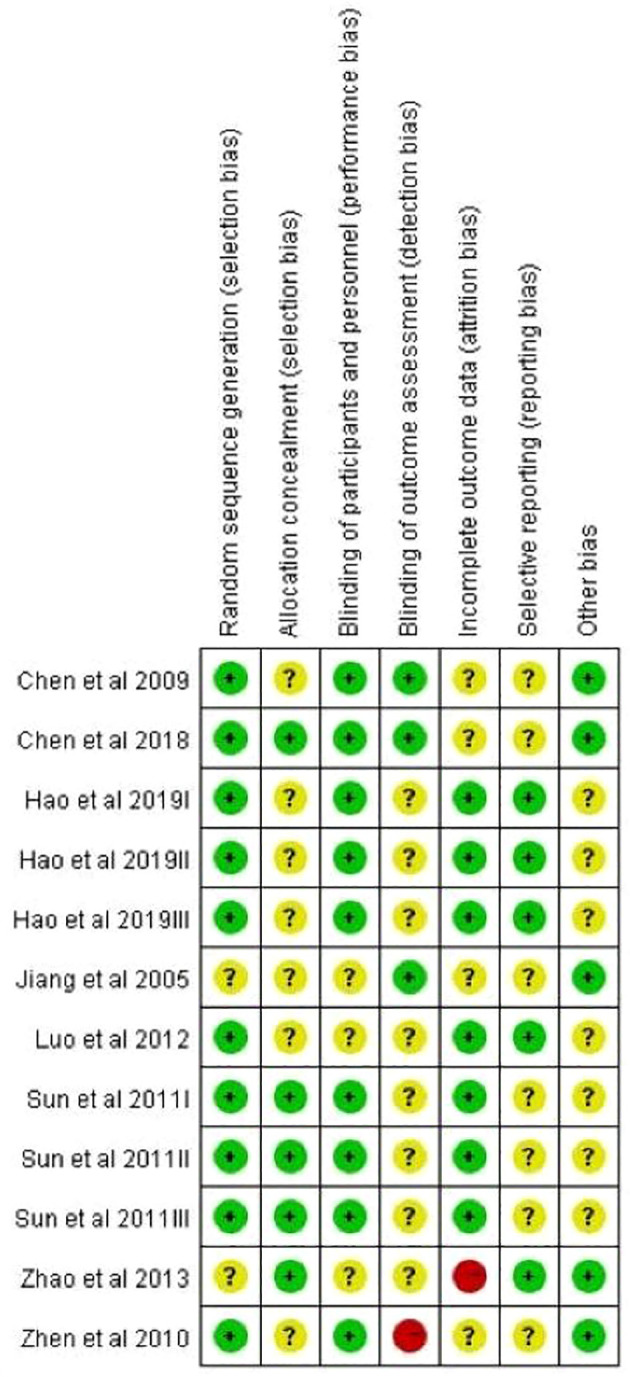
Summary of review authors’ judgements about risk of bias across all domains and studies.

**Figure 2** shows the results of methodological quality evaluation. Due to the inadequate details of the methodological report, 45.2% of the judges among all departments were “the obvious dangers of bias”. Two of the 12 studies had a high risk for at least one department. Low bias risks accounted for 52.4% of all departments.

### Effectiveness of intervention for BMI

**Figure 3:**
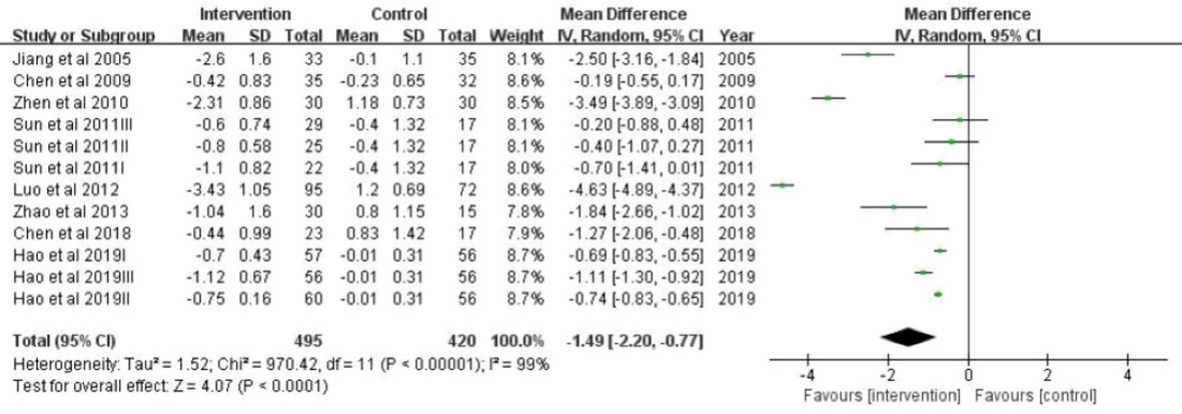
A forest plot providing data on the impact of lifestyle interventions on BMI with 95% confidence intervals of individual studies. Using a random effects model and an inverse variance method, a summary estimate of the 95% confidence interval was calculated. SD, standard deviation; IV, inverse variance; CI, confidence interval.

The overall effect size of 12 studies that reported BMI as an outcome was summarized in **Figure 3**. All of the 12 studies with BMI as an outcome showed a significant effect in favor of the intervention group. A quantitative synthesis of studies by a total of 915 participants showed significantly reduced BMI compared to the control group(mean difference = -1.49; 95%CI:-2.20~-0.77; P<0.00001). However, substantial heterogeneity was detected among the studies (I2 = 99%; p < 0.0001).

**Figure 4:**
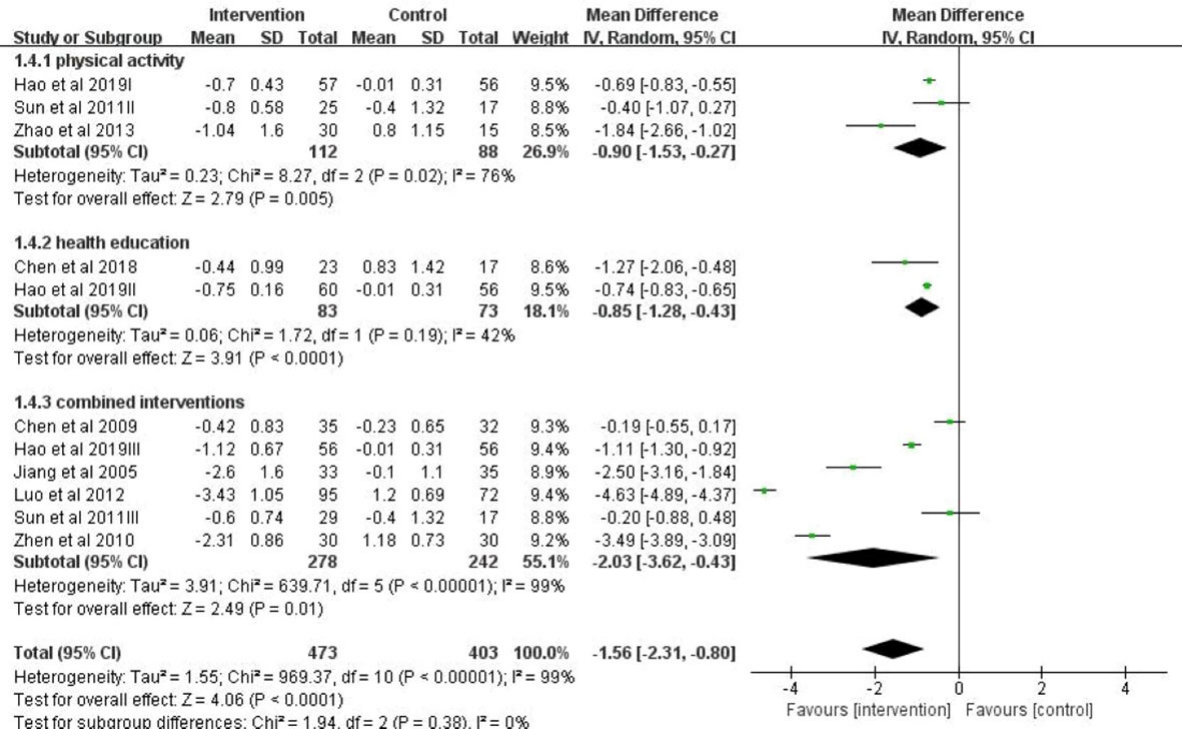
A forest plot providing data on the impact of lifestyle interventions on BMI with 95% confidence intervals of individual studies, grouped by intervention type. Using a random effects model and an inverse variance method, a summary estimate of the 95% confidence interval was calculated. SD, standard deviation; IV, inverse variance; CI, confidence interval.

**Figure 4** showed the results of subgroup analyses that BMI was significantly different by whether or not the studies including multi-component interventions. The effects of lifestyle interventions on BMI change including multiple-component interventions (n = 5) were -2.03 (95% CI: -3.62, -0.43) kg/m^2^, which were stronger than those only with physical activities (n = 3) (pooled BMI = -0.90; (95% CI: -1.53, -0.27) kg/m^2^) or only with health education (n = 2) (pooled BMI =-0.85 (95% CI: -1.28, -0.43) kg/m^2^).

**Figure 5:**
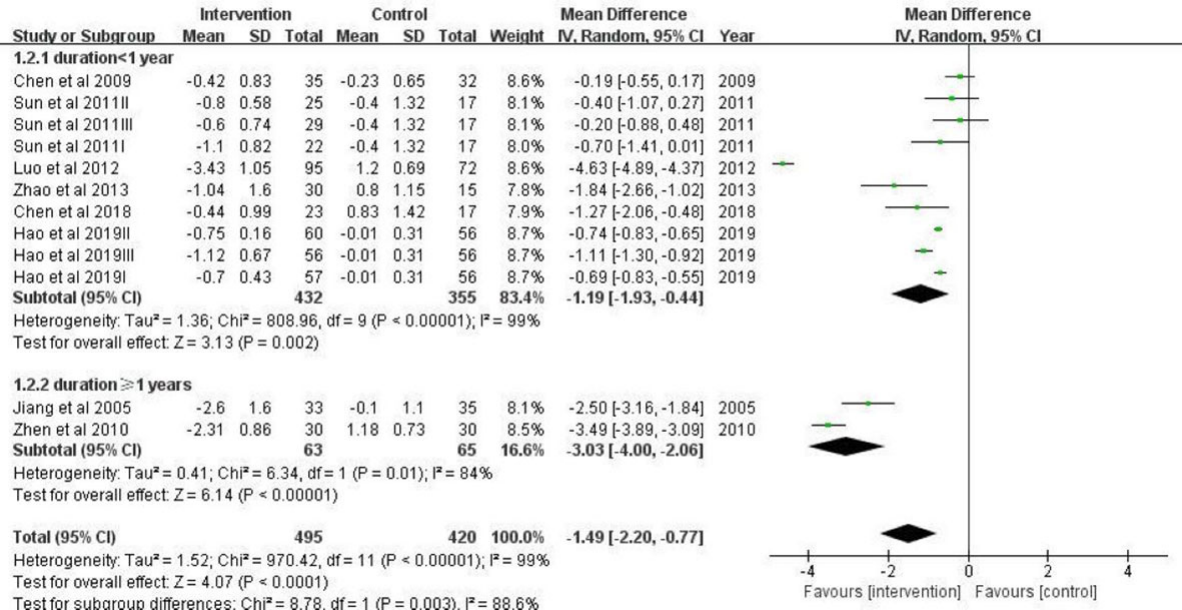
A forest plot providing data on the impact of lifestyle interventions on BMI with 95% confidence intervals of individual studies, grouped by duration of intervention. Using a random effects model and an inverse variance method, a summary estimate of the 95% confidence interval was calculated. SD, standard deviation; IV, inverse variance; CI, confidence interval.

The subgroup analysis also showed significant differences in the BMI change by different duration of intervention (**Figure 5**). When the intervention period was over 1 year (mean difference = -3.03; 95% CI: -4.00, -2.06), this preventive effect was even more remarkable compared with the subgroup with intervention duration <1 year (mean difference = -1.19; 95% CI: -1.93, -0.44).

**Figure 6:**
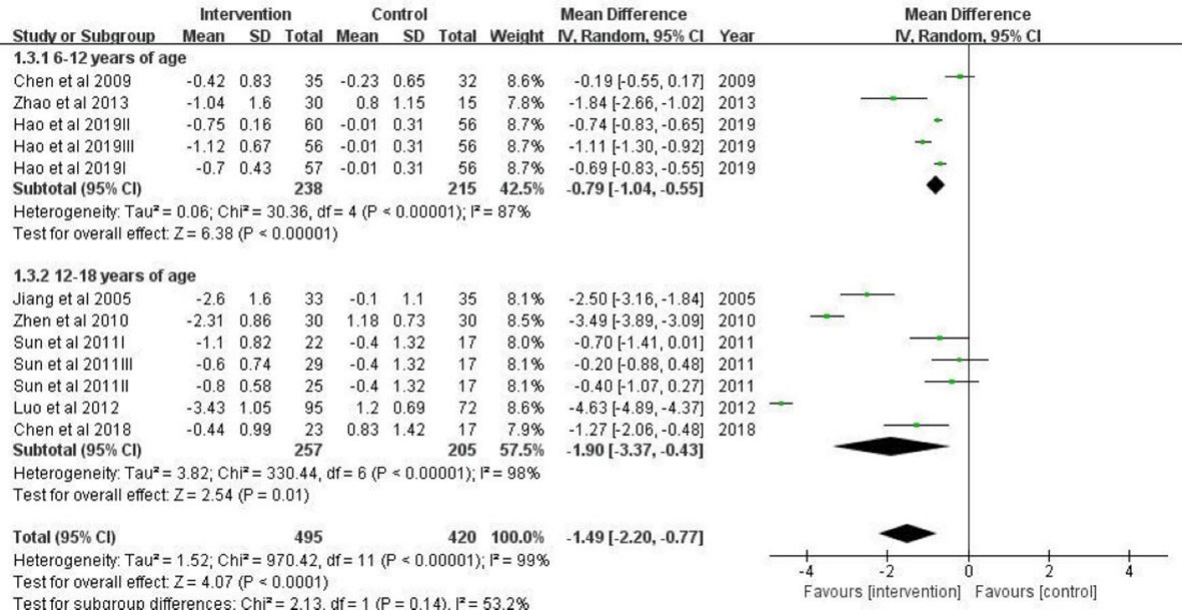
A forest plot providing data on the impact of lifestyle interventions on BMI with 95% confidence intervals of individual studies, grouped by age. Using a random effects model and an inverse variance method, a summary estimate of the 95% confidence interval was calculated. SD, standard deviation; IV, inverse variance; CI, confidence interval.

Grouping estimates revealed that a significant effect of lifestyle intervention on BMI was found in studies that focused on adolescents ≥12 years old (mean difference = -1.90; 95% CI:-3.37, -0.43) (**Figure 6**). In studies conducted in children aged 6-12 years (mean difference = -0.79; 95% CI: -1.04, -0.55), the protective effect was weakened.

### Metabolic consequences

There is a lack of studies assessing the effects of lifestyle interventions on metabolic outcomes in overweight/obese children in China. Only one study reported changes in fasting blood glucose (FBG) (22). This study included a 6-week dietary and physical intervention in 167 Chinese children with obesity aged 11-13 years. This study observed a significant reduction in FBG only in the girls in intervention group, whereas FBG was unchanged in the boys.

Three studies reported lipids profile changes (20–22). Sun et al. reported 240-minute aerobic training per week significantly reduced serum low-density lipoprotein cholesterol whereas no significant effects of interventions on TG and total cholesterol were observed (20). Jiang et al. reported decreases in total cholesterol and triglycerides after a two-year family-based behavior treatment combining a low-calorie diet and PA intervention (21). Luo et al. reported a 6-week energy-limited diet combined with high-intensity aerobic exercise greatly reduced serum cholesterol and TG (22).

Only Chen et al. and Luo et al. reported blood pressure changes (19, 22). Chen et al. observed that both SBP and DBP decreased in the 8-month study, while no significant change in blood pressure levels was found in the control group. This intervention study combined exercise and health education (19). Luo et al. found decreases in both SBP and DBP after the 6-week energy-limited diet combined with high-intensity aerobic exercise intervention (22).

## Discussion

This systematic review identified eight RCTs of lifestyle interventions against overweight/obese Chinese children and adolescents from January 1, 1980, to April 2022. These studies reported BMI as statistically significant and beneficial interventional effects. These findings support proposals that lifestyle interventions be the focus of obesity management.

All of the randomized controlled trials in our study reported the benefits of lifestyle interventions against children overweight/obesity. Regarding the effectiveness of specific intervention components, we found that integrated interventions that combined diet, exercise, and health education were associated with a decrease in BMI. Our findings are unanimous with the proofs from precedent meta-analyses which suggest that multiple lifestyle changes can be beneficial in controlling children’s weight in the short and long term (25–27). However, because the components of lifestyle interventions vary widely in their implementation effectiveness, further exploration of specific interventions is important for improving the effects of metabolism. Evidence also suggests that the longer duration and older child age are associated with favorable weight-loss consequences, which is consistent with our results (28).

Only exercise intervention can lower the BMI but cannot improve metabolic outcomes. Metabolic outcomes are significantly improved only when exercise combines diet or health education. Prevailing recommendations suggest that schoolchildren should receive at least 60 minutes of moderate exercise each weekday (29). In our study, as short as 20 minutes 4 times of exercise each week appears to have admiring outcomes, especially in declining TG and total cholesterol (21). High volume moderate exercise intervention (3 h per session twice each day*6 day weekly) seems to have significantly admiring results in BMI, FBG, TG, and total cholesterol (22). However, the intervention of this intensity is difficult to achieve in family or school, it can only be achieved in weight-loss camp (30).

The types of dietary interventions do not appear to differ significantly in weight loss, consistent with the findings of a recent meta-analysis that regardless of the nutritional composition of an energy-restricted diet, good metabolic benefits can be achieved, which was also found in adult studies.

As far as we know, this is the first systematic review and meta-analysis using subgroup analyses to systematically review recent research, and analyze the effectiveness of lifestyle interventions for overweight/obese children in China. In addition to BMI, we also assessed metabolic outcomes from lifestyle interventions such as FBG, lipids, and blood pressure, which are common methodological limitations of previous reviews (6, 31–33).

Our results have the following limitations. First, there was considerable heterogeneity in the included studies, and this phenomenon is kindly common in complicated obesity interventions. Because of the restricted number of included studies, it is hard to further explore the origins of heterogeneity using meta-regression. Second, given the limited number of included studies as well as the limited sub-group data available for meta-analyses, it is hard to explore whether metabolic outcomes of intervention components were changed by gender, BMI status, or socio-economic factors of the participant’s population.

## Conclusion

From a systematic review and meta-analyses, we found that lifestyle interventions are effective in reducing BMI in Chinese children with overweight/obesity, and the effectiveness is more profound when the lifestyle intervention includes multiple components, lasts longer than one year, and/or is conducted among teens. These findings provide an important evidence base for developing and implementing potentially effective lifestyle interventions for the treatment of overweight/obesity among children in China.

## Data availability statement

The original contributions presented in the study are included in the article/supplementary material. Further inquiries can be directed to the corresponding authors.

## Author contributions

BL and WB conducted the literature search, performed the data extraction, quality assessment and statistical analysis. BL, wrote the original manuscript. ML and SG revised the manuscript. All authors contributed to the article and approved the submitted version.

## Funding

This work was supported by grants from National Natural Science Foundation of China (81970732), Capital’s Funds for Health Improvement and Research (2020-2Z-40117), and the CAMS Innovation Fund for Medical Sciences (CIFMS) (2021-I2M-1-016) and National High Level Hospital Clinical Research Funding (2022-PUMCH-C-014).

## Conflict of interest

The authors declare that the research was conducted in the absence of any commercial or financial relationships that could be construed as a potential conflict of interest.

## Publisher’s note

All claims expressed in this article are solely those of the authors and do not necessarily represent those of their affiliated organizations, or those of the publisher, the editors and the reviewers. Any product that may be evaluated in this article, or claim that may be made by its manufacturer, is not guaranteed or endorsed by the publisher.
